# Disseminated tumour cells as a prognostic biomarker in colorectal cancer

**DOI:** 10.1038/bjc.2011.97

**Published:** 2011-03-29

**Authors:** K Flatmark, E Borgen, J M Nesland, H Rasmussen, H-O Johannessen, I Bukholm, R Rosales, L Hårklau, H J Jacobsen, B Sandstad, K Boye, Ø Fodstad

**Affiliations:** 1 Department of Tumor Biology, Norwegian Radium Hospital, Oslo University Hospital, Oslo 0424, Norway; 2 Department of Surgical Oncology, Norwegian Radium Hospital, Oslo University Hospital, Oslo 0424, Norway; 3 Department of Pathology, Norwegian Radium Hospital, Oslo University Hospital, Oslo 0424, Norway; 4 Institute for Clinical Medicine, University of Oslo, Oslo 0424, Norway; 5 Department of Surgery, Ullevål University Hospital, Oslo University Hospital, Oslo 0424, Norway; 6 Department of Surgery, Akershus University Hospital, Lørenskog 1478, Norway; 7 Department of Surgery, Asker and Bærum Hospital, Sandvika 1336, Norway; 8 Department of Surgery, Aker University Hospital, Oslo University Hospital, Oslo 0424, Norway; 9 Department of Surgery, Diakonhjemmet Hospital, Oslo 0319, Norway; 10 Clinical Trials Unit, Norwegian Radium Hospital, Oslo University Hospital, Oslo 0424, Norway; 11 Department of Oncology, Norwegian Radium Hospital, Oslo University Hospital, Oslo 0424, Norway

**Keywords:** disseminated tumour cells, colorectal cancer, EpCAM, cytokeratin, prognostic biomarker

## Abstract

**Background:**

The study was performed to determine detection rate and prognostic relevance of disseminated tumour cells (DTC) in patients receiving curatively intended surgery for colorectal cancer (CRC).

**Methods:**

The study population consisted of 235 patients with CRC prospectively recruited from five hospitals in the Oslo region. Bone marrow (BM) aspirates were collected at the time of surgery and the presence of DTC was determined by two immunological methods; immunomagnetic selection (using an anti-EpCAM antibody) and immunocytochemistry (using a pan-cytokeratin antibody). Associations between the presence of DTC and metastasis-free, disease-specific and overall survival were analysed using univariate and multivariate methods.

**Results:**

Disseminated tumour cells were detected in 41 (17%) and 28 (12%) of the 235 examined BM samples by immunomagnetic selection and immunocytochemistry, respectively, with only five samples being positive with both methods. The presence of DTC was associated with adverse outcome (metastasis-free, disease-specific and overall survival) in univariate and multivariate analyses.

**Conclusion:**

The presence of DTC was associated with adverse prognosis in this cohort of patients curatively resected for CRC, suggesting that DTC detection still holds promise as a biomarker in CRC.

In colorectal cancer (CRC), treatment decisions are still made almost exclusively based on clinicopathological parameters as described by Dukes almost a century ago (Dukes, 1932), and the search for prognostic biomarkers to improve patient stratification for adjuvant treatment and intensified postoperative surveillance is highly warranted. Despite advances in diagnosis and treatment, a significant proportion (up to 50%) of curatively resected patients develops disease recurrence, primarily as liver and lung metastases (O’Connell *et al*, 2004; Pfister *et al*, 2004). Metastasis development in patients without discernable metastatic disease at the time of primary surgery reflects preceding dissemination of tumour cells with metastatic properties to target organs. Over the last couple of decades, the identification of tumour cells in blood and bone marrow (BM) has been proposed as a potential biomarker of adverse prognosis in solid tumours (Pantel *et al*, 2009). Analyses of tumour cells derived from blood and BM suggest that micrometastases represent a heterogeneous species of cells, possibly not responsive to classic chemotherapeutic strategies. Thus, in addition to being used as a potential biomarker, the possibility of molecular characterisation of the cells might pave the way for therapy specifically targeting such cells, since current treatment options seem to offer limited efficacy with respect to eradicating and controlling this type of disseminated disease.

We previously investigated the presence of disseminated tumour cells in BM (DTC) in 316 patients with assumed CRC using immunomagnetic selection (IMS) with the anti-EpCAM antibody MOC31. Disseminated tumour cells were detected in 17% of patients with CRC with increasing frequency through TNM stages 1–3 (Flatmark *et al*, 2002). In the present work, we present long-term follow-up for this patient cohort, and additionally, we report results obtained by immunocytochemistry (ICC) with anti-cytokeratin antibodies.

## Patients and methods

### Patients

Patients undergoing surgery for assumed or verified CRC were included consecutively from five hospitals in the Oslo region between September 1998 and July 2000. The study was approved by the Regional Ethics Committee (Health Region II, Norway, reference no. S-98080), and patient informed consent was obtained in accordance with the Helsinki Declaration. Bone marrow was collected at primary surgery from both anterior iliac crests from 316 patients. Eighty-one patients were excluded from the analysis, leaving a study population of 235 patients (not invasive cancer (*n*=25); insufficient material for analysis (*n*=2); previous epithelial cancer (*n*=7); histology other than adenocarcinoma (*n*=5); neoadjuvant chemoradiotherapy (*n*=2); incomplete surgical resection (*n*=7); or metastases detected at the time of surgery (*n*=33)). Follow-up data were obtained from consecutive reports from physicians at participating hospitals. Valid observations of the presence or absence of distant metastases required radiological examination. For patients not attending scheduled controls, data were retrieved from patient records or by contacting the patients’ general practitioner. In addition, survival data were obtained from the National Registry of Norway and updated by 1 October 2008. The cause of death was classified as death from CRC, death of other causes or death of unknown cause. For overall survival, median follow-up of patients still alive was 9.3 years (range 8.3–10.2). Histological evaluation of resected specimens was performed in four pathology laboratories, and to ensure consistent staging and grading, one of the study pathologists (JMN) reevaluated reports and primary sections, simultaneously reassessing the presence or absence of lymphocyte infiltration, vascular and perineural invasion and perinodal growth (Table 1).

### Immunomagnetic selection with an anti-EpCAM antibody

Immunomagnetic selection was performed as previously described (Flatmark *et al*, 2002). Briefly, mononuclear cells (MNCs) were separated from BM by gradient centrifugation using CPT tubes (Becton Dickinson Co., Franklin Lakes, NJ, USA), washed once and resuspended in PBS with 1% HSA (Octapharma AG, Ziegelbrücke, Switzerland). Dynabeads M450 rat antimouse IgG1 or M450 sheep antimouse IgG (Dynal, Oslo, Norway) coated with MOC31 antibody (IQ Products, Groningen, The Netherlands) were added to 2 × 10^7^ MNCs at a ratio of one bead to two MNCs in a total volume of 1 ml in 10 ml, round-bottomed test tubes. For control experiments, uncoated beads were used. Samples were incubated at continuous rotation at 4°C for 30 min, and the suspension was then exposed to a strong magnet, and the supernatant was decanted off. Fractions of the remaining cell suspension were examined in a light microscope for the presence of rosetted cells. A sample was classified as positive if a minimum of two cells rosetted at least five beads with the MOC31 antibody, and no rosettes were observed with the control beads.

### Immunocytochemistry with anti-cytokeratin antibodies

Direct cytospins were prepared by centrifuging MNC isolated from BM onto glass slides using a Hettich cytocentrifuge (Tutlingen, Germany). The slides were air-dried overnight and stored at −80°C. Immunocytochemistry was performed as described previously by incubating four slides (2 × 10^6^ MNC) with anti-cytokeratin monoclonal antibodies AE1 and AE3 (Sanbio, Uden, The Netherlands) and the same number of slides were incubated with an isotype-specific irrelevant control antibody (MOPC-21; Sigma Chemical Co., St Louis, MO, USA). Slides were evaluated by light microscopy and cytokeratin-positive cells were subjected to strict morphological classification by a pathologist (EB) according to standardised criteria (Borgen *et al*, 1999; Fehm *et al*, 2006).

### Statistical analysis

Associations between the presence of DTC and clinicopathological variables were tested using two-tailed Fisher's exact test or linear-by-linear association *χ*^2^-test. Univariate survival analysis was performed according to the Kaplan–Meier method, and survival was compared using the log rank test. Multivariate analysis was conducted using the Cox proportional hazards regression model with backward, stepwise elimination of variables. Survival was measured from the date of surgery until death for overall and disease-specific survival, and from the date of surgery until diagnosis of distant metastasis for metastasis-free survival. Data analysis was performed using SPSS version 16.0 (SPSS Inc., Chicago, IL, USA). *P*-values <0.05 were considered statistically significant.

## Results

### Patient characteristics and outcome

Clinical and histological parameters of the study population are summarised in Table 1. Mean age at the time of surgery was 72 years (range 35–98 years). Twenty-nine patients in TNM stage 3 (40%) and two patients in stage 2 (2%) received adjuvant chemotherapy. Tumour perforation at surgery occurred in three rectal cancer patients (two with stage 2 and one stage 3 tumour), and adjuvant radiotherapy was given in these cases. Only 8 of the 69 patients with DTC received adjuvant chemotherapy. Outcome parameters are presented in Table 2. Fifty-five patients developed distant metastases during follow-up (after median 15 months, range 1–59 months), 31 of which had colon cancer and 24 had rectal cancer. A total of 51 CRC-related deaths were registered, and 48 of these patients died of metastatic disease, whereas 3 died of local recurrence. (A discrepancy is apparent between present results and published data (Flatmark *et al*, 2002), as two IMS negative patients previously incorrectly registered as TNM stage 2 have been corrected to TNM stage 1 (as these corrections represented changes at the decimal level, no erratum was published).)

### Detection of DTC

Using the IMS method, EpCAM-positive tumour cells were detected in 41 BM samples (17%) with increasing frequency through TNM stages 1–3; 10%, 18% and 22%, respectively. A median number of 8 tumour cells (range 2–120) were detected in positive samples. Cytokeratin-positive cells were detected in 28 samples (12%) with relatively low detection rate (8%) in TNM stage 2, whereas 14% and 17% of samples were positive in stages 1 and 3, respectively. The median number of tumour cells detected by ICC was 1 (range 1–30), and in 21 of the 28 positive samples only one tumour cell was observed. Of note, only five patients were positive with both methods (one in TNM stage 2 and four in stage 3 patients). The presence of DTC was not associated with any of the clinicopathological variables studied (Table 3).

### Clinicopathological parameters and patient outcome

In univariate analysis, TNM stage, lymph node metastases, tumour localisation in the rectum, absence of tumour lymphocyte infiltration and presence of vascular invasion were associated with metastasis development (Table 4). The presence of lymph node metastases was the main contributor to the prognostic impact of TNM stage, which was also associated with disease-specific and overall survival, while pT-stage was not associated with any of the included outcome parameters. The prognostic relevance of tumour localisation was confined to the distinction between colon and rectum. No significant prognostic differences were observed between the presence of left-sided and right-sided tumours (data not shown). Lymphocyte infiltration, vascular invasion and perineural invasion were associated with disease-specific survival but not with overall survival.

### Detection of DTC and patient outcome

**Figure 1:**
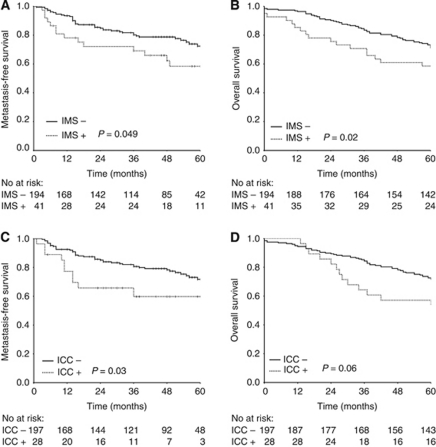
Kaplan–Meier survival plots demonstrating the association between the presence of disseminated tumour cells in bone marrow and survival end points. Immunomagnetic selection (IMS) with an anti-EpCAM antibody and (**A**) metastasis-free (**B**) overall survival. Immunocytochemistry (ICC) with anti-cytokeratin antibody and (**C**) metastasis-free and (**D**) overall survival.

**Figure 2:**
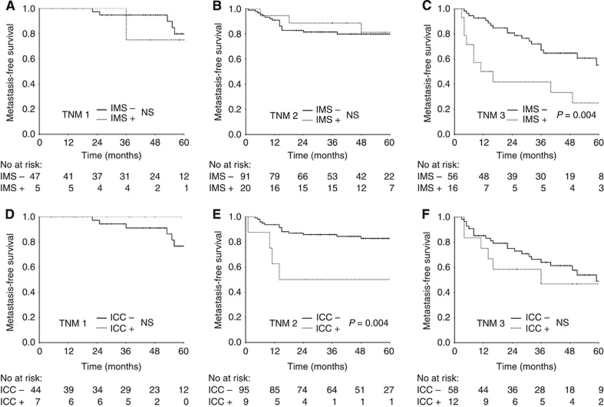
Kaplan–Meier survival plots demonstrating the association between the presence of disseminated tumour cells in bone marrow and metastasis-free survival in stage subgroups at diagnosis. Immunomagnetic selection (IMS) in TNM stages 1 (**A**), 2 (**B**) and 3 (**C**). Immunocytochemistry (ICC) in TNM stages 1 (**D**), 2 (**E**) and 3 (**F**).

The detection of tumour cells in BM was associated with adverse prognosis in this cohort of CRC patients (Figure 1; Table 4). The presence of EpCAM-positive cells, as detected by IMS, was inversely associated with metastasis-free, disease-specific and overall survival in univariate analyses (*P*-values 0.049, 0.03 and 0.02, respectively). Immunocytochemistry detection of cytokeratin-positive cells was inversely associated with metastasis-free and disease-specific survival, but not with overall survival (*P*-values 0.03, 0.002 and 0.06, respectively). In multivariate Cox regression analyses, the impact of detecting EpCAM-positive cells in BM remained evident for metastasis-free survival (Table 4), as well as for disease-specific (*P*=0.002; HR=3.0; CI=1.5–5.8) and overall survival (*P*=0.006; HR=1.9; CI=1.2–3.0). For cytokeratin-positive cells, an association was preserved in multivariate analysis only for disease-specific survival (*P*=0.01; HR=2.5; CI=1.2–5.0). Additional analyses were then performed to assess whether the influence of DTC had particular relevance in classic prognostic subgroups defined by the TNM staging system. In univariate analyses, the prognostic significance of IMS-detected EpCAM-positive cells was evident in TNM stage 3, whereas significant impact of ICC-detected cytokeratin-positive cells was confined to TNM stage 2 (Figure 2).

## Discussion

The main finding of this prospective study was that the presence of DTC was a prognostic biomarker in this cohort of curatively resected patients with CRC. Surprisingly, the use of IMS with an anti-EpCAM antibody and ICC with anti-cytokeratin antibodies for detection of tumour cells resulted in minimal detection overlap, although results obtained with each method were associated with outcome in distinct prognostic subgroups of CRC patients.

The detection rate of EpCAM- and cytokeratin-positive samples in this study (in 17% and 12% of cases, respectively) was somewhat lower than previously reported. A recent meta-analysis identified only six studies that investigated the relevance of DTC in relation to robust outcome parameters, such as metastasis-free and overall survival (Rahbari *et al*, 2010). Of these studies, five utilised ICC and one RT–PCR detection strategies in cohorts comprising 61–167 patients, and DTC were detected in 24–61% of cases. Although suggesting a clinical relevance in CRC, the predictive value of DTC could not be unambiguously determined based on the results reported in these studies. The present work comprises one of the largest CRC patient cohorts examined for the presence of DTC, employing in parallel two distinct detection methods. Although applying the combined results of two detection methods, only 21 of the 55 patients that developed overt metastases could be identified. Conversely, 46 of the 180 patients that did not develop metastatic disease had DTC (combining results from the two methods), giving test specificity and sensitivity for prediction of metastasis development of 74% and 38%, respectively. This infers that in more than half of the metastatic patients, tumour cells were either not present in BM at diagnosis, or EpCAM and cytokeratin expression levels were below the detection limit of the respective method. The finding that detected cells in many cases did not give rise to secondary tumour formation is in turn not surprising, given the known inefficiency of the metastatic process (Chambers *et al*, 2002).

The high detection rate of DTC remains in intriguing contrast to the relatively low incidence of overt bone metastases in CRC (Chambers *et al*, 2002) (in the present study bone metastases were reported in 3 of 55 patients with metastatic disease), suggesting that BM may mirror dissemination of tumour cells to typical metastatic sites (liver and lungs) or alternatively, represent a reservoir of tumour cells able to initiate growth in secondary organs. The kinetics of overt metastasis development (here observed to occur at median 15 months, range 1–59 months after primary surgery) further suggests that DTC may be representative of dormant tumour cells with the ability to escape dormancy control to self-renew and differentiate upon receiving an appropriate stimulus (McGowan *et al*, 2009).

While analysis of cytokeratin expression was chosen primarily for being a hallmark of epithelial cellular origin, EpCAM expression might be of additional biological interest. Complementing its functions as a homeotypic cell adhesion molecule in epithelial-derived cells, EpCAM has been identified as a mitogenic signal transducer and a stem cell marker (Maetzel *et al*, 2009; Munz *et al*, 2009). Thus, detection of EpCAM-positive tumour cells could signify the presence of cells with stem-like properties in BM, potentially contributing to the observed association with metastasis development and patient survival. Interestingly, administration of adjuvant chemotherapy did not influence metastasis development and survival in this patient cohort (data not shown). Although the study design could explain lack of power to detect differences that might still be present, the data may also reflect that the chemotherapy regimen used in the adjuvant setting at that time (5-fluorouracil and calcium folinate) may have been inefficacious. It remains of vital importance to identify and characterise cells responsible for metastatic relapse at the molecular level, with the potential reward of identifying strategies to target therapy towards the prolongation of dormancy or to attain cell death (Epstein, 2005).

The low degree of positive overlap observed between the two detection methods are in accordance with a recent study of 391 early breast cancer patients, in which positive diagnostic overlap of only 3.2% was observed when ICC was performed using two different pan-anti-cytokeratin antibodies, and distinct clinical implications were associated with each antibody (Effenberger *et al*, 2011). In our study, the presence of EpCAM- and cytokeratin-positive cells were prognostic predictors in separate subgroups of CRC patients, in TNM stages 3 and 2, respectively. It is difficult to provide a biological explanation for this finding, and a likely cause could be the low number of cases remaining for calculations in the subgroup analyses. Substantiating the findings of these clinical trials are studies characterising single or small numbers of DTC reporting considerable heterogeneity with respect to genotypic and phenotypic properties of cells from the same individual, reflecting the heterogeneity of the primary tumour (Gangnus *et al*, 2004). Thus, a possible explanation for the lack of overlap between methods could be the presence of subpopulations of tumour cells in BM with distinct molecular characteristics, and to our knowledge, this is the first report to demonstrate clinical impact of such findings in CRC.

Even counting for publication bias, the number of reports supporting a clinical relevance for DTC in solid tumours is remarkable, comprising most solid tumour forms (Pantel *et al*, 2009). Thus, it seems probable that tumour cells are detected, although for most analytical methods, actual proof cannot be provided for each detected cell. The present results add to the evidence suggesting a role for DTC detection as a biomarker in CRC. However, although biologically interesting, the variable results obtained with different detection methods raise more questions than are answered, rendering available technology immature to the extent that it cannot be implemented in routine clinical practice at present. Emerging results from characterisation studies as well as from clinical trials like our own suggest that a detection method suitable for clinical use should be able to account for the apparent heterogeneity of DTC and its relevance in the clinical setting. To address these questions, DTC detection using a range of detection methods should be included in future clinical trials, preferably in the adjuvant setting. This would allow assessment of predictive robustness on a larger scale, determination of potential associations with treatment response as well as facilitate studies of relevant subpopulations of tumour cells with the possibility of correlating results to clinical parameters.

## Figures and Tables

**Table 1:** Baseline clinicopathological characteristics of the study cohort

	**Patients**	
**Parameter**	**Number**	**%**
Total	235	—
		
*Gender*		
Female	106	45
Male	129	55
		
*TNM*		
1	52	22
2	111	47
3	72	31
		
*pT*		
1	8	3
2	49	21
3	154	66
4	24	10
		
*pN*		
0	163	69
1	47	20
2	25	11
		
*Differentiation*		
Well	7	3
Intermediate	203	86
Poor	25	11
		
*Tumour localisation*		
Colon	160	68
Rectum	75	32
		
*Lymphocyte infiltration*		
High	28	12
Intermediate	152	65
Low	52	22
ND	3	1
		
*Vascular invasion*		
Absent	189	80
Present	45	19
ND	1	0.4
		
*Neural invasion*		
Absent	215	92
Present	19	8
ND	1	0.4
		
*Perinodal growth* a		
Absent	30	42
Present	42	58

Abbreviation: ND=not done.

a Perinodal growth was assessed in node-positive patients only.

**Table 2:** Detection of disseminated tumour cells and patient outcome parameters

	**Patients**	
	**Number**	**%**
*EpCAM-positive cells (immunomagnetic selection)*		
Negative	194	83
Positive	41	17
Positive samples TNM stage 1	5	10
Positive samples TNM stage 2	20	18
Positive samples TNM stage 3	16	22
		
*Cytokeratin-positive cells (immunocytochemistry)*		
Negative	197	84
Positive	28	12
ND	10	4
Positive samples TNM stage 1	7	14
Positive samples TNM stage 2	9	8
Positive samples TNM stage 3	12	17
		
*Distant metastases*		
Yes	55	23
No	180	77
		
*Local recurrence* a		
Yes	10	4
No	225	96
		
*Death*		
Yes	113	48
No	122	52
		
*Cause of death*		
Colorectal cancer	51	22
Other	33	14
Unknown	29	12
		
*Location of metastases* b		
Liver	33	
Lungs	26	
Other location	21	

a Six patients had isolated local recurrence.

b Metastases in more than one location were diagnosed in 22 patients.

**Table 3:** Associations between detection of disseminated tumour cells in bone marrow and clinicopathological parameters

	***P*-value^a^**	
	**IMS**	**ICC**
Gender	0.61	1.0
TNM	0.08	0.49
pT	0.29	0.88
pN	0.08	0.37
Differentiation	0.81	0.28
Tumour localisation	0.36	0.19
Lymphocyte infiltration	0.77	0.15
Vascular invasion	1.0	0.11
Neural invasion	0.21	0.71
Perinodal growth^b^	0.57	0.34
IMS	NA	1.0
ICC	1.0	NA

Abbreviations: ICC=immunocytochemistry; IMS=immunomagnetic selection; NA=not applicable.

a *P*-values are from Fisher's exact test or linear-by-linear association *χ*^2^-test.

b Perinodal growth was assessed in node-positive patients only.

**Table 4:** Survival analyses of clinicopathological parameters and disseminated tumour cells in bone marrow

	**Univariate analysis (*P*-values, log rank test)**			**Multivariate Cox regression analysis^a^**		
	**Metastasis-free survival**	**Disease-specific survival**	**Overall survival**	***P*-values**	**Hazard ratio**	**95% CI**
Gender	0.39	0.18	0.25			
*TNM*	<0.001	0.001	0.04	0.03		
1						
2					1.7	0.6–4.3
3					3.0	1.2–7.6
pT	0.06	0.06	0.20			
pN	<0.001	<0.001	0.002			
Differentiation	0.20	0.20	0.45			
*Tumour localisation*	0.02	0.10	0.13	0.02		
Colon						
Rectum					2.1	1.1–3.8
*Lymphocyte infiltration*	0.01	0.008	0.18	0.002		
High						
Intermediate					7.2	1.0–53.5
Low					13.0	1.7–99.3
*Vascular invasion*	0.01	0.03	0.18	0.009		
Absent						
Present					2.3	1.2–4.4
*Neural invasion*	0.11	0.02	0.50			
Absent						
Present						
Perinodal growth^b^	0.41	0.06	0.20			
*Immunomagnetic selection*	0.049	0.03	0.02	0.007		
Negative						
Positive					2.4	1.3–4.5
*Immunocytochemistry*	0.03	0.002	0.06			
Negative						
Positive						

Abbreviation: CI=confidence interval.

a Stepwise Cox regression analysis is shown for metastasis-free survival.

b Perinodal growth was assessed in node-positive patients only.
